# Cold‐adapted Ligninolytic Nanozyme Unveiling New Horizons in Black Liquor Treatment Under Mild Condition

**DOI:** 10.1002/exp2.70237

**Published:** 2026-10-07

**Authors:** Qing Tian, Huile Liu, Yao Chen, Xiaohe Miao, Rong Yang, Yangjun Guo, Mato Knez, Xiyun Yan, Kelong Fan, Lianbing Zhang

**Affiliations:** ^1^ School of Life Sciences and Technology Northwestern Polytechnical University Xi'an China; ^2^ Nanozyme Laboratory in Zhongyuan Henan Academy of Innovations in Medical Science Zhengzhou China; ^3^ Instrumentation and Service Center For Physical Sciences Westlake University Hangzhou China; ^4^ CIC NanoGUNE San Sebastian Spain; ^5^ IKERBASQUE Basque Foundation for Science Bilbao Spain; ^6^ Fac Phys Univ Rijeka Rijeka Croatia; ^7^ CAS Engineering Laboratory For Nanozyme Key Laboratory of Biomacromolecules (CAS) CAS Center for Excellence in Biomacromolecules Institute of Biophysics Chinese Academy of Sciences Beijing China

**Keywords:** black liquor, cold‐adapted, lignin degradation, nanozyme, oxidase

## Abstract

Kraft black liquor, characterised by high lignin content, poses significant challenges for efficient treatment under mild conditions. This study unveils a pioneering solution via a novel cold‐adapted ligninolytic nanozyme constructed from an amorphous manganese‐containing metal‐organic framework (aMnMOF). The aMnMOF features low‐coordination Mn within mixed‐valence trinuclear Mn‐O clusters integrated with a long‐range disordered MOF architecture. This unique design significantly lowered the reaction energy barrier of the rate‐determining step to an exothermic process, endowing it with broad‐spectrum oxidase‐like activities over a wide temperature range (4°C–37°C) without additional oxidants. Based on these advancements, aMnMOF serves not only as a superior nanozyme for oxidative degradation of monomeric, dimeric and complex lignin compounds, but also as a sustainable pathway for real kraft black liquor treatment even under cold conditions, underscoring its superiority over existing methods. This innovative discovery not only advances the rational design of cold‐adapted nanozymes but also provides a significant breakthrough in the field of industrial wastewater treatment.

## Graphical abstract

This work reports a cold‐adapted manganese‐based nanozyme with broad‐spectrum oxidase‐like activity, capable of efficiently degrading lignin and treating kraft black liquor at low temperatures without external oxidants, offering a sustainable and energy‐efficient strategy for industrial wastewater remediation.

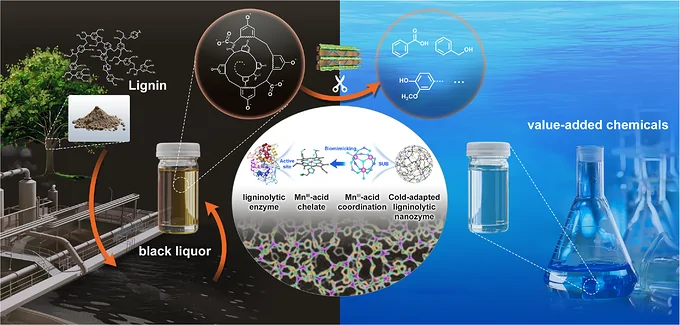

## 1 Introduction

Kraft black liquor, a highly alkaline wastewater with a foul odour, dark colour, low biodegradability and high loading of lignin, lignin derivatives and toxic chemicals, such as chlorinated phenols and unsaturated aromatic compounds, poses substantial environmental risks to aquatic ecosystems [1, 2, 3, 4]. With the annual global production exceeding 1.3 billion tons, improper management of this complex effluent remains a critical challenge. Various physicochemical methods, including incineration, electrocoagulation and noble‐metal‐catalysed advanced oxidation processes, have been developed for lignin degradation and black liquor treatment [5, 6, 7, 8]. However, these approaches are frequently associated with high energy consumption, numerous secondary hazardous substances and compromised cost‐effectiveness [9, 10, 11]. In contrast, biocatalytic approaches utilising microbes and extracellular enzymes demonstrate high efficiency under mild conditions, yet their practical application is severely constrained by the inherent alkalinity, viscosity and elevated solid content of black liquor [12, 13]. Moreover, enzymatic activities exhibit severe temperature sensitivity, becoming largely ineffective during cold seasons or in low‐temperature regions due to activity suppression.

The emerging field of nanozymes, representing nanomaterials with intrinsic enzyme‐like activities, offers innovative alternatives to natural enzyme substitutes by virtue of high efficiency, good stability at both physiological and extreme conditions and easy to obtain with batch production and low cost [14, 15, 16, 17]. Through directed engineering strategies that guide rational design, numerous nanozymes with diverse enzyme‐like properties and superior activity, even surpassing the protein counterparts, were developed [18, 19, 20, 21]. Of particular note is our previous discovery of the first cold‐adapted nanozyme, which presents exceptional and stable activity across both psychrophilic and mesophilic temperature regions, from 0°C to 45°C [22]. This finding not only brings up a novel family of nanozymes that overcome the application limitations of both natural and conventional artificial enzymes, typically restricted to a narrow temperature range, but also provides a new perspective for addressing the aforementioned challenges [23, 24, 25, 26]. Given the distinctive challenges posed by black liquor, in which lignocellulose constitutes the major recalcitrant component, nanozymes with dual functionalities of lignin degradation and cold‐adapted enzyme‐mimicking activity offer a promising solution.

In nature, the microorganisms achieve effective lignin degradation through the secretion of abundant extracellular enzymes, such as manganese peroxidase (MnP), dye‐decolourising peroxidase and versatile peroxidase [7, 27, 28, 29]. These enzymes rely on the generation of Mn^3+^ as a redox mediator, which is stabilised via chelation with organic acids (e.g., oxalate), forming Mn(III)‐O coordination units that serve as the active intermediates responsible for robust oxidative degradation performance [27, 30]. Inspired by this mechanism, metal‐organic framework (MOF) featuring metalloenzyme‐like organic‐inorganic coordination structures, particularly those with Mn(III)‐acid coordination units, should be promising nanozymes as direct oxidative system towards degradation of a variety of substrates [31, 32, 33, 34, 35]. Such an architecture can bypass the requirement for external oxidants to convert Mn^2+^ to Mn^3+^ in natural systems, while simultaneously exhibiting additional enzymatic activities (for instance laccase), owing to the catalytic versatility of Mn‐based nanozymes [36, 37]. Beyond this, with the assistance of amorphous engineering, both enzymatic performance and cold adaptivity can be further enhanced owing to the introduction of abundant open and mixed‐valence metal sites, defective or unsaturated coordination environments and a disordered framework [32, 38, 39, 40, 41, 42, 43]. These characteristics synergistically endow amorphous manganese‐containing MOFs (aMnMOFs) with dual functionalities of ligninolytic and cold‐adapted enzymatic activities, rendering them viable and robust catalysts for efficient black liquor treatment under mild operational conditions.

Drawing inspiration from our design, aMnMOF was prepared by adjusting the nucleation and growth rates, featuring a highly coordinated unsaturated Mn(III)‐O unit coupled with long‐range disorder. The enzymatic activity assay revealed its robust oxidation capability towards a series of model compounds, including phenolic monomers, *β*‐O‐4 bond‐containing dimers and dealkali lignin, in a wide temperature region (4°C–37°C). This demonstrates the superiority of aMnMOF over its natural counterparts, enabling it a substantial avenue to treat black liquor with high efficiency even under cold conditions, with a remarkable reduction in lignin content, chroma, pH and COD (chemical oxygen demand).

## 2 Results and Discussion

### 2.1 Synthesis and Characterisation of aMnMOF

According to our design, a mixed‐valence O‐bridged trinuclear Mn cluster was constructed as the second building unit of the aMnMOF (Figure 1A), which was realised using a Mn^3+^‐containing precursor and a high ratio of organic ligands to suppress the disproportionation reaction of Mn^3+^. To successfully prepare aMnMOF, both hydrothermal and atmospheric heating methods were employed to gain an in‐depth understanding of crystal nucleation and growth kinetics.

**FIGURE 1:**
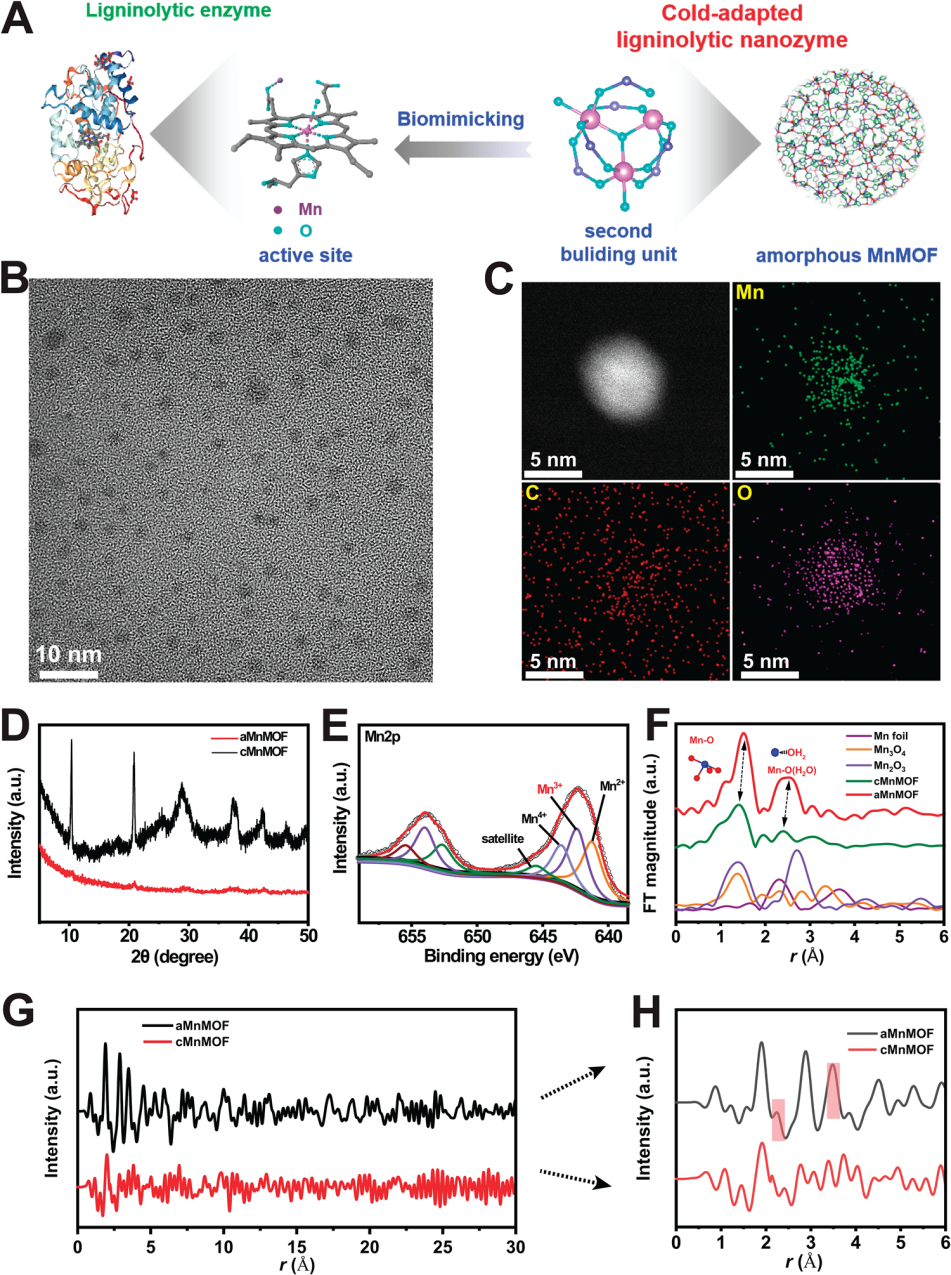
(A) Scheme for aMnMOF design. (B) HRTEM and (C) HADFF images with the corresponding EDS mapping. (D) XRD and (E) XPS pattern (Mn2p), (F) the Fourier transform magnitude of k3‐weighted Mn EXAFS spectra. Radical distribution functions obtained over a wide region (G) and short region (H).

As evidenced by transmission electron microscopy (TEM) (Figures 1B and S1a,b) and X‐ray diffraction spectroscopy (XRD) results (Figures 1D and S2), the materials synthesised via the atmospheric method at temperatures ranging from 50°C to 120°C formed small nanoparticles with gradually enhanced crystallinity. The broad XRD peaks coupled with the absence of lattice fringes in the aberration‐corrected high‐angle annular dark‐field scanning TEM images (Figure 1C) confirmed that the sample obtained at 50°C exhibited typical characteristics of amorphous samples. From the perspective of crystalline strength and lattice structure, the aMnMOF is distinctly different from our previously reported nMnBTC [27, 28, 29]. Notably, no product was obtained at temperatures below 50°C, while a higher temperature of 150°C, though unsuitable for atmospheric reactions in water/ethanol solvents, yielded a mixture of nanoparticles and nanorods (Figures S1d and S2). In contrast, the hydrothermal method produced only nanorods (Figures S1e–g and S2), a typical morphology of crystallised MnMOF (cMnMOF), regardless of temperature. These findings indicate that the nucleation and growth of nanomaterials were controlled at a slow rate under atmospheric heating at low temperatures, thereby enabling the successful synthesis of amorphous aMnMOF.

The chemical states and coordination environment of the Mn centre were comprehensively characterised using X‐ray photoelectron spectroscopy (XPS) and X‐ray absorption fine spectra. As revealed by the detailed deconvolution of the Mn3s (Figure S3) and Mn2p3/2 bands (Figure 1E), aMnMOF exhibited a mixed‐valence state, featuring the coexistence of Mn^4+^, Mn^3+^ and Mn^2+^. Theoretically, the incorporation of Mn^4+^ should induce coordination distortion due to charge imbalance, potentially leading to the generation of oxygen vacancies [44]. This is corroborated by the O 1s XPS spectrum of aMnMOF (Figure S4), where a new peak at 531.7 eV emerges compared to that of cMnMOF. Furthermore, the Mn X‐ray absorption near‐edge structure spectra displayed an edge profile located between those of the reference compounds Mn_2_O_3_ and MnO_2_ (Figure S5), indicating that the average Mn valence state in aMnMOF lies between 3+ and 4+. The Fourier transform magnitude of the Mn EXAFS spectra, representing the average radial distribution of neighbouring atoms, was also analysed. The best fit of the nearest neighbour shell of Mn within cMnMOF aligns well with the reported Mn‐oxo crystallographic structure [45], involving two distant oxygen atoms from one absorbed water molecule at 1.90 ± 0.01 Å and five carboxyl ones at 2.24 ± 0.02 Å (Figure 1F). In contrast, the oxygen neighbours in the first shell of aMnMOF differ in both distance and coordination number, with 1.3 H_2_O molecules at 1.9 ± 0.01 Å and 4.7 atoms at a longer distance of 2.3 ± 0.01 Å. This can be attributed to the highly distorted configuration of Mn in aMnMOF, characterised by relatively weak and unsaturated coordination, which, as reported, significantly enhance both substrate adsorption and transformation [46]. Additionally, the three equal Mn‐Mn neighbours in Mn‐oxo isosceles was twisted, with two distant Mn neighbours around each Mn cation: on average, 1.3 atom at 3.33 Å and 0.7 atoms at a longer distance of 3.46 Å. These structural differences were further confirmed by X‐ray scattering analysis. The pair distribution function spectra of aMnMOF revealed longer Mn‐O (2.3 Å) and Mn‐Mn (3.46 Å) bonds, reaffirming the distortion in Mn‐oxo trimmers. (Figure 1G) Notably, no significant patterns were observed beyond 6 Å in aMnMOF (Figure 1H). The peak intensity, representative of the atomic pair density at specific radial distances [39], exhibited a higher intensity for aMnMOF at short‐range distances (< 6.0 Å). This is consistent with their intrinsic structural characteristics, suggesting a higher intensity of the proximal atomic configurations of aMnMOFs than that of cMnMOFs. Collectively, these results demonstrate the successful preparation of amorphous aMnMOFs, as intended.

### 2.2 The Oxidation of Mono‐Phenolic Substrates by aMnMOF

It is expected that aMnMOFs will be distinguished by substrate diversity, encompassing both phenolic and non‐phenolic compounds. To evaluate this capability, a representative laccase‐like reaction in lignin degradation was initially employed, using 2,4‐dichlorophenol (2,4‐DP) as the reductive substrate and 4‐aminoantipyrine (AP) as the chromogen. As illustrated in Figure 2A, aMnMOF exhibited exceptional phenol oxidase‐like activity, transforming the solution colour from colourless to red. In contrast, cMnMOF induced almost no observable colour change under the same conditions and measurable activity appeared only when both the substrate and nanozyme concentrations were increased and the reaction time was extended (Figure S6). A comparative analysis among the samples prepared under various conditions revealed that the nanozyme activity was inversely correlated with the crystalline intensity, with the maximum activity observed for aMnMOF (Figure S7). Kinetic analysis revealed that aMnMOF demonstrated a comparable *K_m_* value but a higher *V*_max_ relative to its natural counterpart (Figure S8), indicating good substrate binding affinity and superior catalytic efficiency. Further assays conducted on aMnMOF at gradually decreasing temperatures revealed an almost negligible activity loss from 40°C to 4°C (Figure 2B), underscoring its robust tolerance to low temperatures and remarkable adaptability across both psychrophilic and mesophilic conditions. Additionally, the aMnMOF exhibited broad substrate compatibility toward diverse phenolic compounds (Figure 2C), a feature highly consistent with the natural laccase's ability to accommodate structurally complex lignin‐derived substrates. Meanwhile, it retained exceptional stability under harsh operational conditions, including a wide pH range, elevated temperatures, high salinity, repeated catalytic cycles (Figure S9) and long‐term storage (Figure S10). These collective advances suggest that aMnMOF is a promising alternative for overcoming the limitations of its natural lignin‐degrading enzymes, which typically require multiple‐step oxidation and the assistance of an oxidant cofactor to achieve Mn(III). Furthermore, its efficiency was superior to that of most of the reported laccase‐mimicking nanozymes (Table S1). Consequently, aMnMOF was selected for further investigation.

**FIGURE 2:**
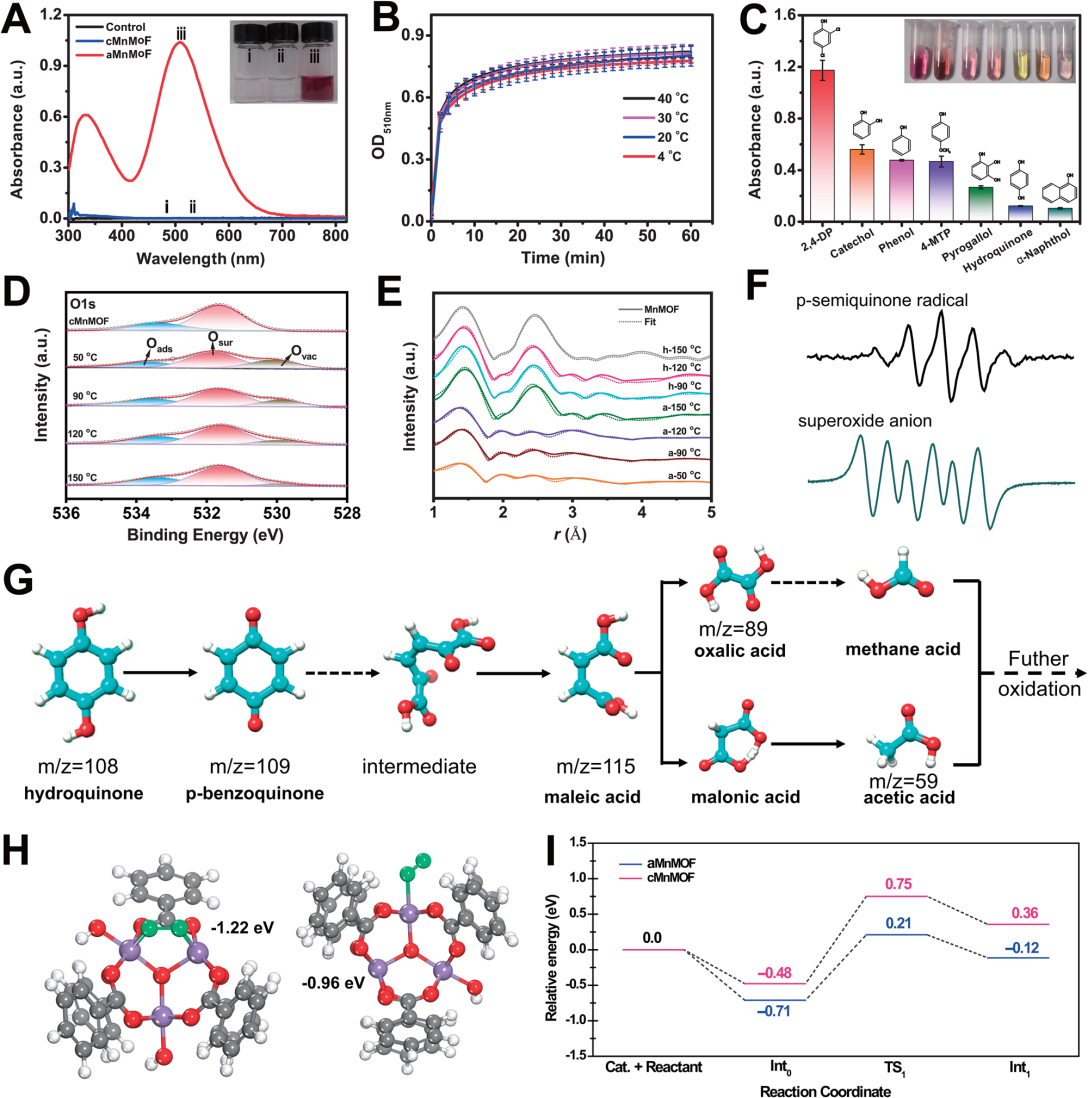
(A) UV‐vis adsorption curve and (B) enzymatic kinetic curve of the reaction system. (C) Oxidation towards different phenolic substrates. (D) The O1s XPS spectra and (E) Fourier transform magnitude of the k3‐weighted Mn EXAFS spectra. (F) The ESR spectra. (G) Reaction pathway for hydroquinone oxidation by aMnMOF. (H) Structural model of O_2_ adsorption on aMnMOF (left), cMnMOF (right) and the corresponding *E*_ads_. The Mn, O and C atoms are represented by purple, red and grey balls, respectively. The adsorbed O_2_ is marked by green atoms to make a distinction. (I) Gibbs free energy diagram of the H abstraction reaction from hydroquinone.

### 2.3 Catalytic Mechanism of aMnMOF for Monophenol Oxidation

To clearly identify the active sites of the aMnMOF, we conducted a detailed structural comparison of the as‐synthesised samples. According to the XPS results, the lower‐crystalline samples exhibited a higher proportion of high‐valence Mn (Mn^3+^ and Mn^4+^) along with an increased average oxidation state (AOS, Figure S3, Table S2), as calculated from the multiple splitting widths of Mn3s (Δ*E*_Mn3Ss_). This was accompanied by an increase in the number of oxygen vacancies induced by charge imbalance (Figure 2D). As demonstrated in the literature, Mn(III) species possess robust oxidative potential towards various structures [47], which should be further enhanced by Mn^4+^ incorporation via the oxygen vacancy and rapid redox cycling between Mn^3+^‐Mn^4+^/Mn^2+^‐Mn^3+^ pairs. The fitting data for the local neighbourhood of Mn revealed that weakly crystalline samples exhibited pronounced structural distortions, featuring longer Mn‐O distances (≈2.2 Å) in the first coordination shell and reduced coordination numbers with decreasing crystallinity. These characteristics likely create more accessible active centres for substrate and oxygen adsorption, facilitating rapid electron transfer over short diffusion paths. In contrast, rod‐like samples with high crystallinity displayed a saturated coordination structure similar to that of cMnMOF (Figure 2E), directly evidencing the suppression of coordination unsaturated sites. Additionally, their specific surface area and pore structure were examined. The results show that both parameters increase with crystallinity, where aMnMOF possesses the smallest surface area and pore size and cMnMOF the largest. (Figure S11 and S12). This trend arises from the structural evolution from short‐range disordered frameworks to well‐ordered porous networks. These results exclude the possibility that the superior catalytic activity of aMnMOF originates from surface area or pore size effects. Therefore, it can be proposed that the combination of Mn^3+^ and highly unsaturated coordination centres constitutes the critical active sites responsible for the excellent cold‐adapted nanozyme activity of the aMnMOF.

Subsequently, an in‐depth investigation was conducted to elucidate the catalytic mechanism of aMnMOF. To precisely delineate the reaction steps, hydroquinone was employed as the model substrate to simplify the reaction system. As evidenced by the electron paramagnetic resonance data, both p‐semiquinone and superoxide anion (·O^2−^) radicals were generated in the reaction system (Figure 2F). Further quenching experiments using 2,2,6,6‐tetramethylpiperidine‐1‐oxyl (TEMPO) as a scavenger of ·O^2−^ showed a slight inhibitory effect on activity (Figure S13a), whereas the addition of semiquinone, an analogue of p‐semiquinone, significantly enhanced the activity (Figure S13b). These findings indicate that both p‐semiquinone and·O^2−^ are the active intermediates responsible for the oxidation reaction. Based on the analysis of reaction intermediates monitored by mass spectroscopy (Figure S14), a reaction pathway was proposed, as shown in Figure 2G. Initially, hydroquinone is converted to a phenoxy radical, which typically exists in various mesomeric forms, such as a carbon‐centred radical in the quinone ring. This radical subsequently reacts with O^2−^ to form an unstable ether peroxide, which undergoes spontaneous ring fission and transforms into an aliphatic structure (a muconic acid derivative) with a free carboxylic group. Notably, the·O^2−^ in this system was derived from the intrinsic oxidase‐like properties of aMnMOF (Figure S15), unlike the MnP‐like reaction, in which it is released through the chelation of Mn^3+^ with organic acids in the presence of O_2_. The aMnMOF system then acts again, attacking the remaining aliphatic radicals, which undergo further oxidation reactions, likely forming low‐molecular‐weight organic acids.

Based on these results, density functional theory calculations were conducted to theoretically elucidate the catalytic mechanism. Given the low‐coordination Mn (*n* = 4.7) in aMnMOF, a MnO_4_ model was constructed as a tri‐Mn configuration featuring oxygen coordination numbers of 4, 5 and 6. This is distinguished from the typical MnO_5_ model of cMnMOF, which contains two Mn atoms coordinated to six oxygen atoms and one Mn atom bonded to five oxygen atoms (Figure S16). As illustrated in Figure s and S17, aMnMOF exhibits significantly stronger adsorption interactions with hydroquinone (−0.71 eV) and O_2_ (−1.22 eV) compared to those of cMnMOF (−0.48 eV and −0.96 eV, respectively). This could be attributed to the abundance of open sites for substrate anchoring. Additionally, the Gibbs free‐energy diagram (∆*G*) for each step in the reaction profile of H atom abstraction from hydroquinone to generate semiquinone was derived. The rate‐determining step was identified as TS1 (Figure S18), owing to its larger energy barrier within the entire process. Notably, aMnMOF requires less energy (0.92 eV) to achieve this step than cMnMOF (1.23 eV). Importantly, aMnMOF significantly reduced the energy required for the H abstraction reaction, transforming the endothermic process observed in cMnMOF (∆*G* = 0.36 eV) into a favourable exothermic process with a ∆G of −0.12 eV (Figure 2I). From both thermodynamic and kinetic perspectives, this finding aligns well with the characteristics of cold‐adaptive enzymes and low‐temperature catalysts ([44]), providing robust evidence for the exceptional oxidase‐like activity of aMnMOF at low temperatures.

The aforementioned findings enable us to delineate the reaction mechanism as follows (Figure 3): (1) the binding of phenolic molecules, such as hydroquinone, to the active sites of aMnMOF; (2) the subsequent oxidation by Mn(II) leading to the formation of semiquinone radicals and the reduction of Mn(III) to Mn(II) through electron transfer; (3) the activation of O_2_ to generate·O_2_^−^, which facilitates further oxidation, ring opening of the phenolic compound and eventual conversion into small organic molecules; (4) the conversion of O_2_^−^ to H_2_O and O_2_, coupled with the oxidation of Mn(II) back to Mn(III), thereby completing the catalytic cycle of the aMnMOF nanozyme.

**FIGURE 3:**
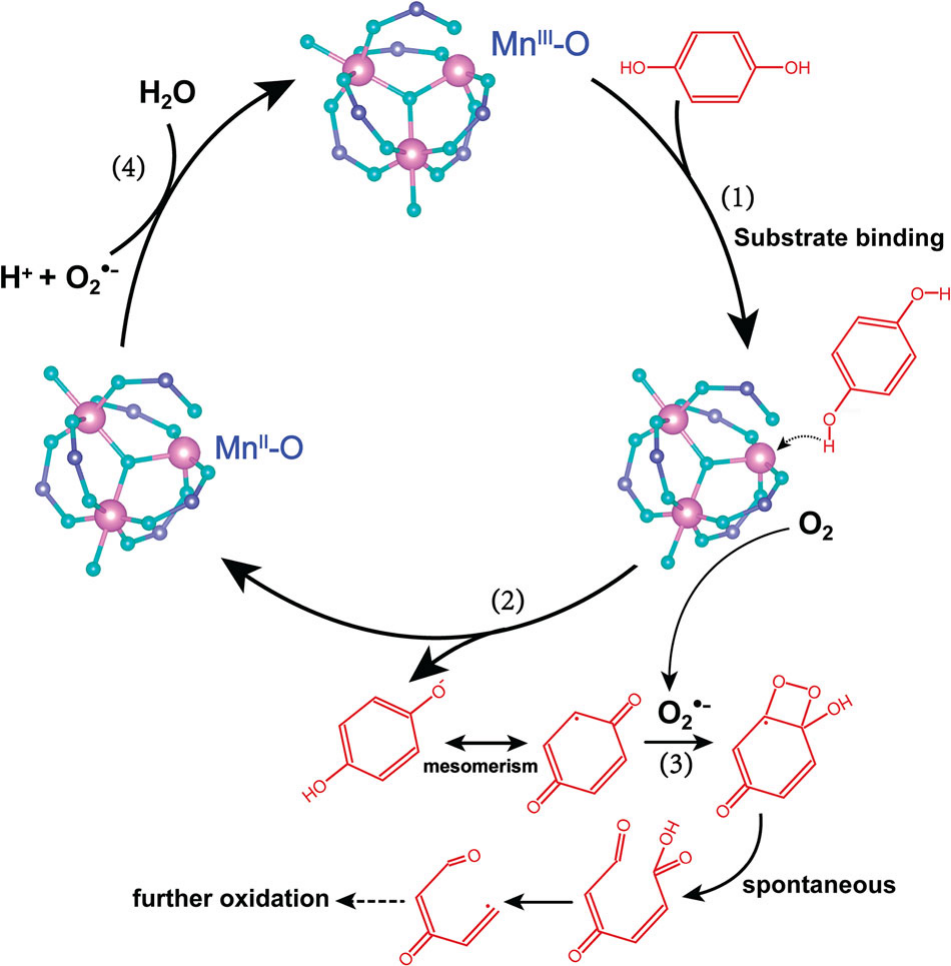
Scheme of the phenol oxidation reaction catalysed by aMnMOF.

### 2.4 Degradation of Dimer‐Phenolic Compounds and Dealkali Lignin

Given that the aromatic substructures in lignin are predominantly interconnected through *β*‐O‐4 linkages, we systematically investigated the catalytic performance of dimers containing *β*‐O‐4 bonds using both phenolic and non‐phenolic moieties, represented by guaiacylglycerol‐*β*‐guaiacyl ether (GGE) and phenoxy‐1‐phenylethanol (PPE) (Figure 4A). Catalytic evaluation revealed that the aMnMOF efficiently cleaved the *β*‐O‐4 bond in PPE, with phenol and acetophenone identified as the primary products (Figure 4B). Similarly, the GGE‐aMnMOF system demonstrated successful C‐O bond scission, yielding guaiacol as the characteristic cleavage product (Figure 4C). To validate the catalytic mechanism of the aMnMOF for *β*‐O‐4 bond cleavage, the radicals in these systems were measured. As shown in Figure S19a, alkoxy and carbon radicals were detected in the aMnMOF‐GGE system. This implies that the model compound, when oxidised by aMnMOF, was first converted to the C_α_ radical. This radical readily undergoes rearrangement to form alkoxy radicals, which further propagate fragmentation via hydrogen abstraction, enabling *β*‐O‐4 bond cleavage and formation of carbonyl compounds. In the aMnMOF‐PPE system, the capture of phenoxy radicals and alkoxy radicals (Figure S19b) indicates cooperative pathways for *β*‐O‐4 bond cleavage. The phenoxyl radical generated via H‐abstraction from phenolic‐OH groups directly induce heterolytic C‐O bond cleavage, yielding aromatic aldehydes and carbocation intermediates. While the alkoxy radical promotes homolytic *β*‐scission, breaking the C‐O bond into phenol and acetophenone [48]. Notably, comparable substrate conversion efficiencies were observed for both systems at 4°C and 37°C (Figure S20), demonstrating the exceptional cold‐adapted catalytic capacity of aMnMOF for dimeric substrate oxidation. This temperature‐independent performance profile surpasses that of conventional catalytic systems that typically require a noble metal catalyst and heating treatment, as evidenced by the comparative data in Table S3. The observed catalytic efficiency is particularly significant, outperforming native ligninolytic enzymes, which generally require multi‐enzyme synergy or exogenous co‐oxidants to facilitate reaction progression.

**FIGURE 4:**
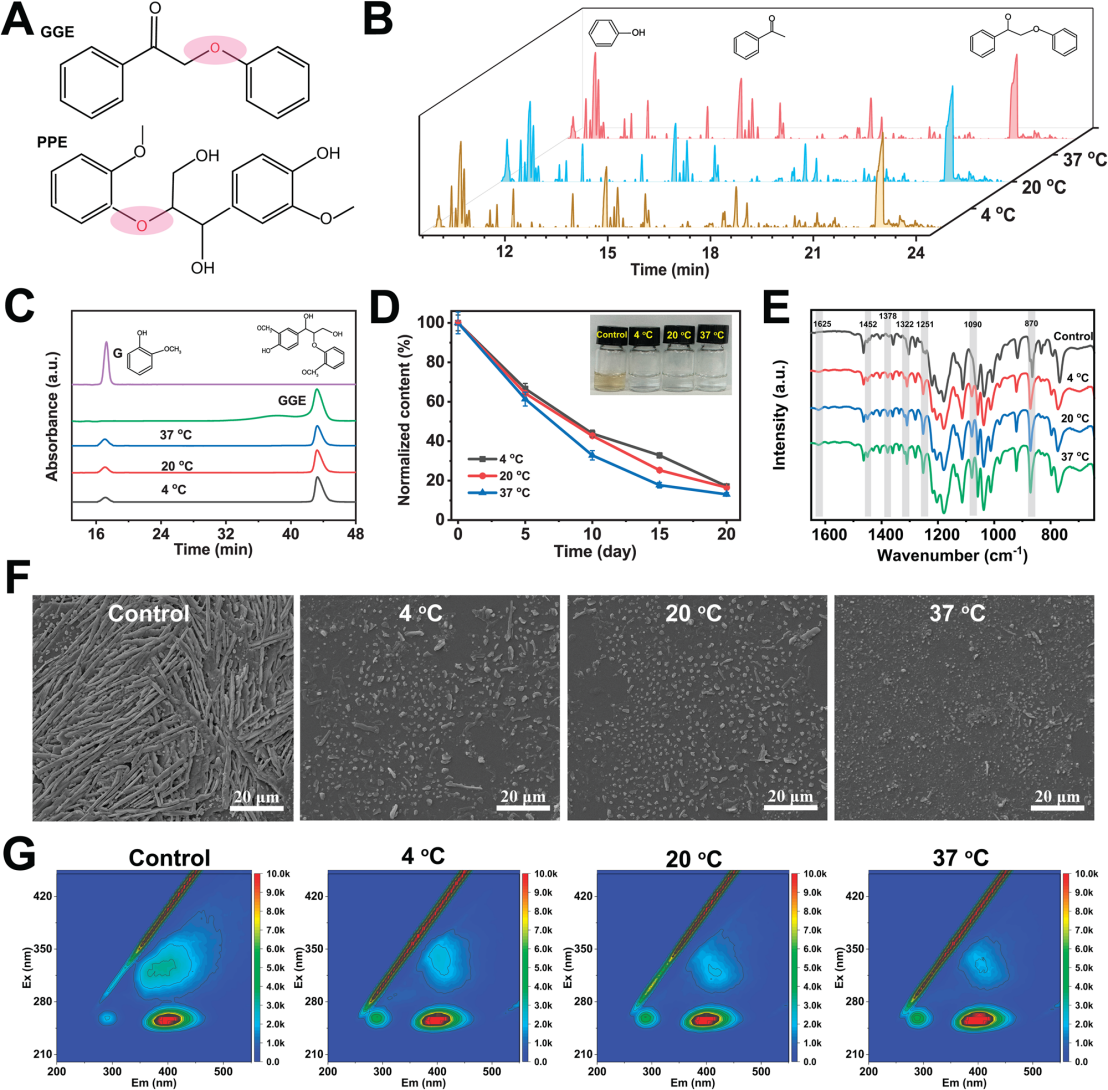
Degradation of dimers and dealkali lignin compounds. (A) Structure of PPE and GGE. (B) GC‐MS of the PPE‐aMnMOF system. (C) HPLC results of GGE degradation. (D) Normalised dealkali degradation rate at different temperatures and the corresponding reaction images (inset). (E) FTIR spectra, (F) SEM images and (G) EEM results of the dealkali lignin system before and after the treatment.

Further investigations were conducted to evaluate the degradation capability of the aMnMOF towards dealkali lignin, which is a recalcitrant byproduct of kraft pulp. After treatment, the UV‐vis spectra analysis revealed progressive attenuation, evidenced by a significant reduction in the characteristic wavelengths of 280 nm and 340 nm (Figure S21), corresponding to the π→π* transition of aromatic moieties and conjugated C*_α_* = C*_β_* bonds in lignin macromolecules, respectively. Notably, comparable degradation efficiencies were achieved across various temperatures (37°C, 20°C and 4°C), as indicated by the colour change from light yellow to near‐colourless solutions (Figure 4D). Complementary FTIR analysis (Figure 4E) depicted substantial peak intensification within the wavenumber range of 1625 to 870 cm^−1^, which are detailed in Table S5 and demonstrate the cleavage of interconnected lignin skeleton into fragmented components. Specifically, the disappearance of the phenyl ring vibrational signals indicates the scission of conjugated aromatic systems, which is a characteristic hallmark of lignin depolymerisation. The disappearance of phenolic‐OH groups suggests the oxidative conversion of phenolic moieties into quinone derivatives or progressive degradation into aliphatic intermediates. Furthermore, the concomitant emergence of a distinct peak at 2926 cm^−1^, corresponding to ‐CH_2_ groups, provides additional evidence for lignin degradation. Morphological alternations observed through SEM (Figure 4F) clearly show the structural disintegration of lignin from its initial rod‐like architecture to irregular particulate debris, indicative of macromolecular framework collapse.

Three‐dimensional excitation‐emission matrix fluorescence spectroscopy (3D‐EEM), which is sensitive and selective for the detection of dissolved organic compounds [49], provides additional evidence of structural transformations. As shown in Figure 4G, the original lignin exhibited two characteristic fluorescence signatures: a humic‐like peak (*E_x_/E_m_* = 235–255/280–320 nm) and a fulvic acid‐like signal (*E_x_/E_m_* = 235–255/390–450 nm). Following aMnMOF treatment under all tested temperature conditions (4°C, 20°C and 37°C), the humic‐like peaks were significantly enhanced, whereas the fulvic acid‐like signals markedly decreased. This pattern suggests that lignin underwent decomposition and subsequent transformation into more stable humic acid‐like substances. These spectral evolutions collectively demonstrate the depolymerisation of dealkani lignin into monomeric aromatic species over a wide temperature range. As established by enzymatic mechanisms, lignin preferentially undergoes phenol oxidation and *β*‐O‐4 bond cleavage under enzymatic attack. These reactions trigger autocatalytic radical chain reactions, generating reactive intermediates that cumulatively depolymerise the macromolecular framework of lignin into small organic compounds. Therefore, the aMnMOF plays a key role in initiating lignin degradation in a cold‐adaptive manner, thereby achieving high efficiency across a wide temperature range.

### 2.5 Kraft Black Liquor Treatment by aMnMOF

Based on the discussion above, it is anticipated that aMnMOFs can serve as a promising alternative to tackle the challenges associated with kraft black liquor treatment. The industrial papermaking wastewater used in this study was highly polluted and exhibited strong alkalinity (pH 10.3) and high viscosity (11.5 mPa·s) due to the abundance of lignin and organic matter. In addition, its chroma and chemical oxygen demand (COD) greatly exceeded the upper detection limits of the analytical instruments (Table S6); therefore, the samples were diluted 100‐fold for accurate determination of both parameters. The aMnMOF treatment led to a pronounced decolourisation effect, changing the colour of the raw sample from tan to light yellow (Figure 5A), while the diluted sample shifted from light brown to nearly transparent (inset of Figure 5B). This phenomenon is primarily attributed to the efficient decomposition of lignin, as evidenced by the sharp decline in UV‐vis absorbance at 280 nm (Figures 5B and S21). Concomitantly, substantial reductions in pH (Figure 5C), COD (Figure 5D), chroma and viscosity (Table S6) were observed, collectively confirming the high catalytic efficiency of aMnMOF in black liquor treatment. Specifically, the viscosity decrease reflects the breakdown of solid and high‐molecular‐weight lignin and organic macromolecules into smaller, more soluble fragments. Moreover, comparable results were achieved with gradually decreasing temperatures (from 37°C to 4°C), although there was a slight reduction in the conversion efficiency (Figure 5A–D, Table S6). However, further application at 0°C and below was limited, as the system became frozen under such conditions. The morphological changes were directly identified from the SEM images (Figure 5G). Initially, the raw samples were intact rods covered with a layer of viscous organic matter. Upon treatment, they cracked into small pieces and particles and large pores emerged on the surface of the skeleton. The tolerance of aMnMOF to various temperatures, especially low temperatures, has not been previously achieved using traditional strategies‐further highlights its superior performance under mild conditions. The tolerance of aMnMOF to a wide range of temperatures, particularly low temperatures, which has not been achieved using traditional strategies, highlights its superior performance under mild conditions. Furthermore, aMnMOF maintained excellent treatment efficiency after five consecutive cycles, showing only a slight decrease in chroma and COD removal performance (Figure S22). XRD (Figure S23) and TEM (Figure S24) analyses of the recovered nanozyme confirmed that its morphology and amorphous framework were well preserved. Additionally, the mixed‐valence states and their relative proportions remained stable after the reaction (Figure S25, Table S4). These findings collectively demonstrate the outstanding catalytic stability and durability of aMnMOF.

**FIGURE 5:**
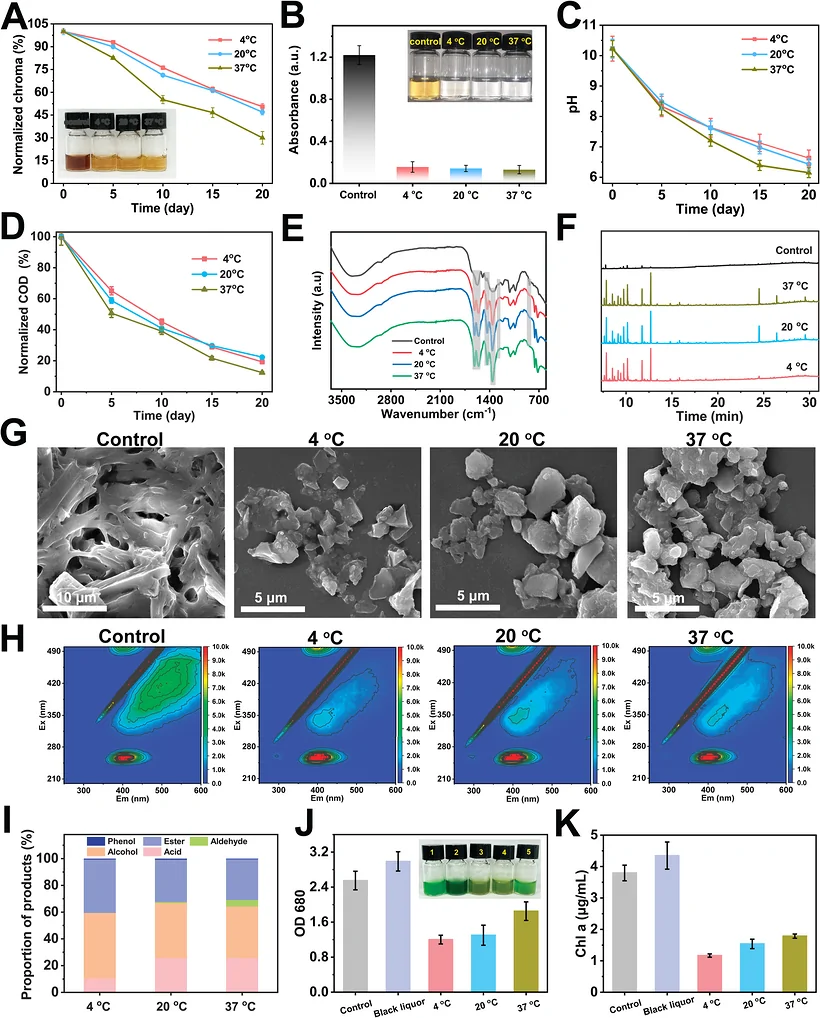
Treatment of black liquor with MnMOF. (A) UV‐vis absorbance, (B) normalised chroma, (C) pH, (D) normalised COD, (E) FTIR spectra, (F) GC‐MS results, (G) SEM images and (H) 3DEEM results of the diluted samples before and after treatment at different temperatures. (I) Product distribution of black liquor after treatment. (J) UV‐vis absorbance and (K) Chl *a* of cyanobacteria solutions with/without treatment; insets are the corresponding images.

Considering the potential of black liquor treatment for producing high‐value chemicals, both FTIR and GC‐MS characterisations were conducted. The FTIR results (Figure 5E) reveal a significant disappearance of characteristic vibrations associated with C‐H deformation (832 and 1475 cm^−1^) and phenyl rings (1462 and 1577 cm^−1^), which are key indicators of lignin depolymerisation, suggesting the cleavage of conjugated aromatic systems. Furthermore, the diminution of phenolic hydroxyl groups (1286 cm^−1^) provides evidence of phenolic moieties in quinones or their further degradation to aliphatic intermediates. These observations coincide with the emergence of aliphatic chain hydrocarbons (1557 and 1614 cm^−1^), along with methyl and methylene groups, which collectively confirm the disruption of rigid aromatic networks into low‐molecular‐weight fragments. GC‐MS measurement (Figure 5F), alongside HPLC characterisation (Table S6), confirmed that the products from the treated samples consisted of a variety of small molecules, mainly classified as acids, alcohols, ketones, alkanes, esters, phenols, aldehydes and aromatics (Figure 5I). The high content of aromatic compounds and organic acids in the products is likely the main cause of the remarkable pH reduction and indicates the possible cleavage of *β*‐O‐4, *α*‐O‐4 and C*_α_*‐C*_β_* bonds, as well as ring‐opening reactions in lignin. These products possess significant potential for high‐value utilisation. For instance, oxalic acid is widely used as a chelating agent in textile and metal‐cleaning industries and ethanol and other low‐molecular‐weight alcohols are valuable intermediates for biofuel and fine chemical production [50]. Owing to the broad‐spectrum oxidation of complex black liquor substrates, the reaction yielded a mixture of multiple products without a dominant target compound, which hinders large‐scale purification and extraction. Enhanced selectivity toward desired products could be achieved through rational catalyst design and reaction‐pathway control. Nevertheless, numerous successful reports on the extraction of similar compounds from black liquor through low‐energy separation processes provide a solid foundation for realising this goal [50, 51]. The structural changes were also evaluated using 3D EEMs. The spectra in Figure 5H exhibit a main peak characteristic of humic and fulvic‐like fluorescent compounds located at 310–490/400–600 nm (*E_x_/E_m_*), which shifted to shorter wavelengths after treatment. The fluorescence intensity of this peak declined sharply following the aMnMOF treatment, indicating the degradation of complex aromatic lignin derivatives. Concurrently, the emergence or intensification of shorter‐wavelength peaks at 240–320/280–350 nm (*E_x_/E_m_*) suggests the accumulation of simpler, less conjugated products such as acids or aliphatic intermediates [52]. These spectral shifts confirm the fragmentation of high‐molecular‐weight lignin polymers into smaller oxidised species, which is consistent with the ability of aMnMOF to break C‐O/C‐C bonds and oxidise aromatic rings. These findings clarify the superiority of aMnMOFs over both natural systems which are highly temperature‐sensitive and often ineffective under cold conditions and catalytic system (for instance Fenton reaction, light‐driven MnO_2_ system) typically featured with high energy consuming (Table S8) [53]. Moreover, aMnMOFs achieve efficient treatment without the need for additional oxidants, which is a requirement for many natural enzymes, thereby simplifying the treatment process and reducing costs. In addition, the simple and scalable synthesis, inexpensive raw materials and excellent recyclability of aMnMOF further highlight its techno‐economic feasibility, making it a cost‐effective and sustainable alternative for large‐scale black liquor remediation.

Considering that humic substances in wastewater serve as promoters for cyanobacterial proliferation and consequent ecological hazards [54, 55], we systematically investigated the impact of both raw and aMnMOF‐treated black liquor on cyanobacteria growth dynamics. Prior to the experiments, we evaluated the bio‐compatibility of the aMnMOF. The results demonstrated that even at high concentrations (200 g/mL), aMnMOF had a low inhibitory effect on cyanobacterial growth (Figure S26a). Furthermore, potential toxic impacts arising from ion leaching were conclusively ruled out, as evidenced by the comparable growth rates observed in deionised water and supernatant from aMnMOF‐containing aqueous systems (Figure S26b). To eliminate the potential impact of residual aMnMOF, the treated black liquor was centrifuged and microfiltered before collecting the supernatant for cultivation experiments. Comparative analysis suggested that the original black liquor significantly stimulated cyanobacterial biomass accumulation (Figure 5J) and chlorophyll‐a biosynthesis (Chl *a*, Figure 5K), whereas the aMnMOF‐treated sample exhibited a remarkable suppression of cellular density and photosynthetic pigment content. Macroscopic chromatic analysis (inset in Figure 5J) provides direct visual evidence of significant cellular inactivation in the treated samples. This distinct biological response likely stems from the effective removal of humic acid derivatives, which are critical microbial growth substrates during the aMnMOF treatment process. Particularly noteworthy is the maintenance of these inhibitory effects across a wide temperature range, from 37°C to 4°C, presenting a slight reduction in treatment efficiency. Building on these advancements, aMnMOFs demonstrate superior functionality to natural enzymes by exhibiting both high efficiency and cold‐adapted activity without exogenous oxidants, while exhibiting remarkable stability.

## 3 Conclusions

In summary, a novel cold‐adapted ligninolytic nanozyme of aMnMOF was successfully constructed and demonstrated exceptional catalytic performance in the degradation of lignin compounds, encompassing monomolecules, dimers and dealkali lignin, under low‐temperature conditions. Notably, its application in black liquor treatment resulted in a remarkable reduction in the lignin concentration, pH, COD and chroma. Furthermore, the by‐products generated during this process are industrially relevant for high‐value chemical production. This breakthrough highlights the superior efficacy of aMnMOFs over conventional enzymes and existing artificial counterparts, while providing a strategic framework for designing cold‐adapted nanozymes. Specifically, cold‐adapted ligninolytic nanozymes hold promise for black liquor treatment, meeting the standards of operational efficiency, profitability, environmental sustainability and resource circularity.

## Author Contributions

**Qing Tian**: methodology, writing – original draft preparation. **Huile Liu and Yangjun Guo**: data curation, writing – original draft preparation. **Yao Chen**: conceptualisation, writing – reviewing and editing. **Xiaohe Miao**: software. **Rong Yang**: visualisation, Investigation. **Xiyun Yan, Kelong Fan and Lianbing Zhang**: supervision.

## Funding

This work was supported by the following funding: National Natural Science Foundation of China (No.22577102); Nanozyme Laboratory in Zhongyuan Key R&D Program (No. NLZ‐KP2024NIC03); The Chinese Academy of Sciences Project for Young Scientists in Basic Research (No. YSBR‐089).

## Ethics Statement

There is no animal experiment or human subjects/clinical trials involved in this research.

## Conflicts of Interest

Xiyun Yan and Kelong Fan are members of the *Exploration* editorial board and they were not involved in the handling or peer review process of this manuscript.

## Supporting information




**Supporting File**: exp270237‐sup‐0001‐SuppMat.docx.

## Data Availability

The data that support the findings of this study are available from the corresponding author upon reasonable request.
