# Supplementary material for: Cold‐adapted Ligninolytic Nanozyme Unveiling New Horizons in Black Liquor Treatment Under Mild Condition
Source: Exploration (Beijing). 2026 Oct 7;6(5):70237. doi: 10.1002/exp2.70237 (PMC13646180; doi:10.1002/exp2.70237)
Supplement: Supplementary file 1 [file EXP2-6-70237-s001.docx]

**Supplementary Information**

**Cold-adapted Ligninolytic Nanozyme Unveiling New Horizons in Black Liquor Treatment Under Mild Condition**

**Experimental Section**

**Materials**

1,3,5-Benzenetricarboxylic acid (BTC), manganese(II) nitrate tetrahydrate (Mn(NO_3_)_2_·4H_2_O), manganese(III) acetate dihydrate (Mn(CH_3_COO)_3_·2H_2_O), 2-(N-Morpholino) ethanesulfonic acid hydrate (MES), 4-aminoantipyrine, catechol, hydroquinone, 2,4-dichlorophenol, phenol, pyrogallol, α-naphthol, 4-methoxyphenol, acetic acid, sodium acetate, ethanol, methanol, 1-butanol, β-carotene, 3,3',5,5'-Tetramethylbenzidine (TMB), and laccase (≥ 0.5 U/mg) were purchased from J&K Scientific Ltd. (China). Tempol was obtained from Med Chem Express (MCE, USA). 2-Phenoxy-1-phenylethanol (PPE) and guaiacylglycerol-*β*-guaiacyl (GGE) were purchased from Aladdin (China).

**Synthesis of MnMOF**

The crystalline MnMOF (cMnMOF) was synthesized using a standard method. Specifically, 18 mmol of Mn(NO_3_)_2_·4H_2_O and 6 mmol of BTC were dissolved in a 60 mL mixture of DMF and H_2_O (1:1 v/v). After complete dissolution, the solution was transferred into a 100 mL Teflon-lined stainless-steel autoclave and heated at 120 °C for 96 hours. The resulting colorless crystals were collected by centrifugation at 1000 rpm for 5 min, followed by washing five times with deionized water, and dried in a vacuum overnight.

The amorphous MnMOF (aMnMOF) was synthesized by dissolving 4 mmol of Mn(CH_3_COO)_3_·2H_2_O and 1 mmol of BTC separately in a mixed solvent of ethanol and H_2_O. After stirring for 10 minutes, the solutions were combined and heated at various temperatures (50, 90, 120, and 150 °C) under atmospheric pressure. After reaction, the precipitate was washed five times with deionized water, and collected by centrifugation at 8000 rpm for 5 min. For comparison, a hydrothermal method was employed in a Teflon-lined autoclave at temperatures of 90, 120, and 150 °C. In this study, the MOFs prepared under atmospheric conditions are denoted as a-n, while those prepared using the hydrothermal method are labeled h-n, where n represents the temperature.

**Characterizations**

The morphology and microstructure of the MOFs were characterized by using double Cs-corrected transmission electron microscopy (Tecnai G2 F30 S-TWIN, FEI, USA). X-ray diffraction (XRD) patterns were collected using a D8 DISCOVER A25 diffractometer over the 2θ range of 5^o^ to 80^o^ with Cu Kα radiation. Unpaired electrons or free radicals were measured by electron paramagnetic resonance (EPR, EMXPLUS10/12, Germany). Fourier transform infrared (FTIR) spectra were recorded using a Nicolet iS50 Spectrometer (Thermo Fisher, USA) in the range of 4000-600 cm^-1^ with a resolution of 2 cm^-1^. Ultraviolet-visible (UV-vis) absorption spectra and time-dependent absorbance spectra of the reaction system were recorded with a UV-vis spectrophotometer (Nanodrop One, Thermo Scientific, USA) equipped with a temperature-controlled microvolume. High-resolution powder X-ray diffraction and pair distribution function (PDF) scattering systems were used to analyze atomic distances and intensities in the MOF. The surface electronic states of the samples were analyzed by X-ray photoelectron spectroscopy (XPS, Thermo ESCALAB 250 spectrometer, USA) using a monochromatic Al K*α* X-ray source. The Mn XPS spectra were first calibrated to the C1s peak at 284.8 eV. The Mn 3s region was then analyzed to determine the oxidation states by fitting two multiple-split peaks, and the energy separation (ΔE) between them was used to estimate the dominant Mn valence states, based on the established relationship that ΔE≈6.0, 5.0, and 4.6 eV correspond to Mn^2+^, Mn^3+^, and Mn^4+^, respectively. Guided by this analysis, the Mn 2p XPS spectra were further deconvoluted using XPSPEAKER software, applying Tougaard background subtraction, mixed Gaussian-Lorentzian peak shapes, a fixed 11.2 eV spin-orbit separation, and constrained intensity ratios between Mn 2p3/2 and Mn 2p1/2. The relative proportions of the oxidation states were calculated from the integrated peak areas of the fitted components.

**Phenol oxidase-like activity assay**

The phenol oxidase-like activity was measured via a chromogenic reaction of phenolic compounds with 4-AP. The catalyst (2 mg/mL, 10 μL), 4-AP (10 mg/mL, 10 μL), and 2,4-DP (2.5 mg/mL, 20 μL) solutions were mixed with MES buffer (100 mM, pH 6.8, 970 μL) under stirring. The absorption spectrum at 510 nm was measured using a UV-vis spectrophotometer (Nanodrop One) under kinetic model. Similar assays were performed with various analog substrates (phenol, hydroquinone, naphthol, 4-methoxyphenol, pyrogallol, and catechol) following the same procedure. The kinetic parameters were measured by using 4-AP as substrate with varied concentration. Michaelis-Menten constant (*K_m_*) and the maximum reaction rate (*V_max_*), were calculated basing fitted curve. A comparative analysis was conducted by using laccase as control.

**Degradation of hydroquinone**

20 μL hydroquinone solution (2.5 mg/mL) and 10 μL nanozyme (2.0 mg/mL) were mixed into 970 μL deionized H_2_O under stirring, and incubated at room temperature for 1 hour. Then they were centrifuged at 14000 rpm for 10 minutes, and the supernatant was collected after filtering with a 0.45 μm filtration membrane (Amber Laboratory Technology Co., Ltd., China). The collected samples were analyzed by use of liquid chromatography mass spectrometry (LC-MS, Thermo ULTIMATE3000 UHPLC Q EXACTIVE MS). The detection conditions were shown as follows:

Column: InertSustain C18

Column Cat. No: 5020-88026

Eluent: A) CH_3_CN; B) H_2_O

Flow Rate: 1.0 mL/min

Col. Temp: 35 ^o^C

Injection Vol: 10 µL

**Enzymatic performance assay**

The phenol oxidation reaction was conducted at varying pH (from 3.5 to 10) and different NaCl concentrations (0 to 1200 mM). The aMnMOF was incubated at temperatures ranging from 30 °C to 90 °C for 1 hour, followed by activity test at 25 °C. After reaction, the aMnMOF was recovered and assayed again following the same procedure. This process was repeated multiple times to assess the cycling stability of aMnMOF. To evaluated its storage stability, the aMnMOF stored in a sealed bottle was sampled at each week interval for activity measurement.

**Electron spin resonance (EPR) measurement**

EPR was measured using DMPO as a spin-trapping agent. For ·OH determination, 10 μL of aMnMOF (2 mg/mL) was added to 890 μL MES buffer (100 mM, pH 6.8), followed by the addition of 100 μL DMPO (100 mM) under stirring. After 10 minutes of incubation, the EPR spectra were recorded on a Bruker EMX PLUS spectrometer with the following conditions: 100.00 kHz microwave frequency, 6.325 mW microwave power, 1.000 GHz modulation frequency, 1.0 G modulation amplitude, 100.0 G sweep width, 15.0 dB microwave decay, 30.00 s sweep time, and 3502.00 G center field. Semiquinone radicals were measured directly in the reaction system without a trapping agent. In quenching experiments, tert-butyl alcohol, TEMPO, and *β*-carotene were added separately into the reaction system to examine their impact on activity. The radicals in dimmeric compound-aMnMOF systems were directly measured utilizing DMPO as scavenger.

**Density function theory (DFT) calculation**

All DFT calculations were carried out using the CP2K code.^1^ All calculations employed a mixed Gaussian and planewave basis sets. Core electrons were represented with norm-conserving Goedecker-Teter-Hutter pseudopotentials,^2-4^ and the valence electron wavefunction was expanded in a double-zeta basis set with polarization functions^5^ along with an auxiliary plane wave basis set with an energy cutoff of 450 eV. The generalized gradient approximation exchange-correlation functional of Perdew, Burke, and Enzerhof (PBE)^6^ was used. Test calculations showed that the total energy change of the reactive system was negligible (<0.01 eV) when the maximum force convergence criteria of 0.001 hartree/bohr was used. Each reaction state configuration was optimized with the Broyden-Fletcher-Goldfarb-Shanno (BGFS) algorithm with SCF convergence criteria of 1.0×10^-6^ au. To compensate the long-range van der Waals dispersion interaction between the adsorbate and the framework, the DFT-D3 scheme^7^ with an empirical damped potential term was added into the energies obtained from exchange-correlation functional in all calculations. Transition states of elementary steps were located using the climbing image nudged elastic band (CI-NEB) method^8-9^ with seven intermediate images along the reaction pathway between the initial and the final states. Each identified transition state was further confirmed by the vibrational frequency analysis. The reaction energy is calculated as the total energy difference between the final and the initial states. The forward and reverse activation barriers for each elementary reaction are defined as the total energy difference between the initial and transition states and between the final and transition states, respectively.

**Degradation of GGE**

All degradation experiments were conducted in 2 mL centrifuge tubes containing 1 mL of 3 µM GGE solution at varied temperatures (4 °C, 20 °C, and 37 °C). The pH of the reaction system was adjusted to 6.8 with MES buffer (100 mM). Then, 0.2 mg aMnMOF was added to the solution under stirring conditions (250 rpm). Samples were collected at fixed time intervals, followed by filtering with 0.22 μm filter membrane (ANPEL Laboratory Technologies Inc, Shanghai, China), and analyzing with high-performance liquid chromatography (HPLC). The test conditions were set as follows:

System: Waters Alliance HPLC system

Column: InertSustain C18

Column Cat. No: 5020-88026

Eluent: A) CH_3_OH; B) Imidazole buffer; A/B=15/85, v/v

Flow Rate: 0.85 mL/min

Col. Temp: 40 ^o^C

Injection Vol: 80 µL

Sample: Standard

Imidazole buffer: imidazole (68.08 g), disodium ethylenediaminetetraacetate (0.37 g) and magnesium acetate (10.72 g) were mixed and dissolved in 800 mL H_2_O. The pH was adjusted to 7.2 with acetic acid and the volume of solution was fixed to be 1000 mL with deionized H_2_O.

**Degradation of PPE**

All degradation experiments were conducted in 2 mL centrifuge tubes containing 1 mL of 3 µM PPE solution (in MES buffer, 100 mM, pH6.8) at varied temperatures (4 °C, 20 °C, and 37 °C). 0.2 mg aMnMOF was added to this solution under stirring conditions (250 rpm). Samples were collected at fixed time intervals, after filtering with syringe filters membrane (0.22 μm). Then they were treated with anhydrous sodium sulfate to remove excess water and analyzed by gas chromatography/mass spectrometry (GC/MS). The test conditions were set as follows:

System: Thermo TRACE 1300 Gas Chromatograph

Column: HP-5

Injection Vol: 2 µL

Helium gas: He,

Flow rate: 12 mL/min

Column temperature: Initially maintained at 75 °C for 5 min, and then increased to 280 °C for 25.625 min.

Detector: FID 280 °C

Sample: Standard

**Degradation of dealkali lignin**

To examine the degradation efficiency towards dealkali lignin, 1.5 mL of a dealkali lignin solution (0.1 mg/mL in DMSO, in a 2 mL tube) was added with 0.2 mg of aMnMOF and incubated under stirring at varied temperatures (4 °C, 20 °C, and 37 °C). At each time interval, the supernatant was collected for products analysis, which is achieved by centrifugation (14,000 rpm for 10 minutes) and filtered through a 0.45 μm filter membrane. The solid residue was dispersed in ethanol solution, which was then dripped onto silicon wafer with a straw.

The collected supernatant was analyzed with the following procedure. First, the lignin content was assayed by measuring the UV-vis absorbance at 280 nm. The components changes were determined by three-dimensional excitation-emission matrix fluorescence spectroscopy (3D EEMs) measurement using a high-sensitivity fluorescence spectrophotometer (F98, Shanghai Lengguang Technology Co., Ltd.) equipped with a 1 cm quartz fluorescence cell. The operating parameters were set as follows: excitation light source of 150 W xenon arc lamp, PMT voltage at 610 V, automatic response time, slit width of 5 nm, scan speed of 30,000 nm/min, excitation wavelength range (*λ*_Ex_)-200-500 nm, emission wavelength range (λ_Em_)-200-600 nm. The chemical groups were characterized by Fourier-transform infrared (FTIR) spectra on a Nicolet iS50 Spectrometer (Thermo Fisher, USA) in the region 4000-600 cm^-1^ at a resolution of 2 cm^-1^.

**Treatment of black liquor by aMnMOF**

Wood-derived black liquor from Xi'an Paper Mill (China) was used for analysis. Both raw and diluted (100 times with MES buffer (100 mM, pH 6.8)) samples were tested. Typically, 5 mL of the solution mixed with 1 mg of aMnMOF was added to a glass bottle and stirred at 250 rpm. The supernatant of the reaction system was collected each day and filtered through 0.22 μm membrane. The UV-vis spectra (280 nm), pH, chroma, chemical oxygen demand (COD), viscosity, FTIR spectra, GC-MS, and 3D EEMs of the samples were measured. The UV-vis spectra (280 nm), pH, chemical FTIR spectra and 3D EEMs measurements were processed following similar procedures described in dealkali lignin degradation. Specifically, COD was determined using the potassium dichromate method, which is shown as follows: 10 mL of the sample was diluted ten times with deionized water and transferred to a 250 mL round-bottom flask. Then 5.0 mL of a standard potassium dichromate solution (0.25 mol/L) and 15 mL of a sulfuric acid-silver sulfate solution were added into the solution in consequence, followed by gently shaking to ensure thorough mixing. The mixture was reacted for 2 hours under boiling condition. After cooling, 150 μL of ferroin indicator was added and the solution was titrated with a standard ferrous ammonium sulfate solution (0.1 mol/L). The endpoint was identified when the solution color changed from yellow to blue-green and finally to reddish-brown. The volume of the ferrous ammonium sulfate solution (0.1008 mol/L) used was recorded as *V_1_.* Simultaneously, a blank test was conducted following the same procedure except that the sample was replaced with 10.0 mL deionized water. The volume of the standard ferrous ammonium sulfate solution (0.1 mol/L) used in the blank titration was recorded as (*V_0_*). The COD was calculated with the following formula:

$$COD（O_{2}，\text{mg/L）=}\frac{（V_{0}-V_{1}）\times C\times8\times1000}{V}\times n$$

Where:

*n* — dilution times of the raw black liquor,100
*C* — concentration of the standard ferrous ammonium sulfate solution (mol/L)
*V_0_* — volume of the standard ferrous ammonium sulfate solution used in the blank titration (mL)
*V_1_* — volume of the standard ferrous ammonium sulfate solution used in the black liquor titration (mL)
*V* — volume of the black liquor sample (mL)
*8* — 1/2 molar mass of oxygen (g/mol)

The chroma was assessed using the platinum-cobalt colorimetric method (GB 11903-89). The chroma was calculated with the following formula:

$$CU=500\times\frac{A_{1}}{A_{2}}\times\text{n}$$

Where:

*n* — dilution factor of the water sample, 5
 *A_1_* — absorbance of the black liquor
 *A_2_* — absorbance of the 500 CU platinum-cobalt standard solution, 0.12

Chroma removal rate (CU%) was calculated as follows:

$$CU\%=\frac{U_{0}-U_{1}}{U_{0}}$$

Where:
*U_0_* — Chroma before treatment
*U_1_* — Chroma after treatment

Chroma standard stock solution was prepared as follows: 1.245 g of potassium hexachloroplatinate and 1.000 of cobalt chloride hexahydrate were dissolved in 500 mL water. Then, 100 mL of hydrochloric acid (1.18 g/mL) was added, and the solution was diluted to the 1000 mL with water (equivalent to 500 color units).

The viscosity of Kraft black liquor was measured using a rotational rheometer (Brookfield DV3T). Prior to measurement, samples were equilibrated at the target temperature (25-80 °C) using a thermostatic water bath. The apparent viscosity was recorded were conducted under steady-state shear conditions until the readings stabilized, and each test was repeated three times to ensure reproducibility. For GC-MS measurement, 1500 μL ethyl acetate was added to solubilize the hydrolyzation products and derivatized with 500 μL BSTFA (containing 1% TMCS) at 100 ◦C for 1 h. Then they were treated with anhydrous sodium sulfate to remove excess water and tested by GC-MS using an Agilent 7000D gas chromatograph, which is equipped with an Agilent 5975 mass selective detector (MSD) with an Agilent HP-5 ms capillary chromatographic column (30 m, 250 µm, 0.25 µm). The testing parameters and conditions are as follows: HP-5 capillary column was used and heated to 50 °C for 5 minutes, followed by heating to 280 °C with rate of 10 °C/min and held for 5 minutes. The detailed testing parameters are: injector temperature 280 °C, solvent delay time 8 minutes, equilibration time 1 minutes, vacuum time 2.5 minutes, ion source temperature 250 °C, transfer line temperature 200 °C. Mass spectra were recorded in full scan mode from 30 to 550 m/z, and chemical identification was performed using the NIST 2.0 mass spectral database.

**Culture of cyanobacterium**

The cyanobacterium Synechococcus sp. PCC 7942 was obtained from the Institute of Hydrobiology, Chinese Academy of Sciences. The cyanobacteria were cultured aseptically in conical flasks containing BG11 medium in a low-temperature vertical cold light source incubator. The culture temperature was maintained at 30±1°C with a light intensity of 50 μE. Cells in the exponential growth phase were resuspended in fresh BG11 medium at an initial density of ~1.45 × 10^6^/mL. Prior to the experiments, we first evaluated the biocompatibility of the aMnMOF. 2 mL of the suspension was mixed with 200 μL of samples aqueous solution with different concentrations (from 0.01 mg/mL to 5 mg/mL). The cell intensity was detected at each 24 h interval with an UV-vis spectroscopy at 680 nm. To investigate the impact induced by potential ions leaching, the cyanobacterium was cultured with the supernatant of aMnMOF aqueous solution after centrifugation, followed by cell intensity assay after 4 days incubation. To eliminate potential impact caused by residual aMnMOF, the treated black liquor was centrifugated and microfiltered and filtering (using a 0.22 μm filter membrane) for further cultivation experiments. 2 mL of the suspension mixed with 200 μL of black liquor samples (both raw and treated ones with aMnMOF) at varying temperatures (4 °C, 20 °C, and 37 °C). For comparison, a control was cultured with an equivalent volume of deionized water. After incubation for 4 days in a fully illuminated indoor cultivation chamber, the Chlorophyll a (Chl *a*) was extracted from the cells using methanol, and determined by measuring UV-vis absorbance at 665 nm and 652 nm. The concentration of Chl *a* was calculated using the following formulas:

$$\text{Chl }\text{a }\left( \text{mg/mL} \right)\text{=16.72O}\text{D}_{\text{662}}\text{-9.16O}\text{D}_{\text{645}}$$

**Supporting Figures and Tables:**

Figure S1. TEM images of the MnMOFs prepared at varied temperature under atmospheric pressure: (a) a-90 ^o^C, (b) a-120 ^o^C, (c) a-150 ^o^C, and high pressure: (d) h-90 ^o^C, (e) d-120 ^o^C, (f) d-150 ^o^C.

Figure S2. XRD spectra of the as-prepared MnMOFs.

Figure S3. The Mn3S xps spectra of the samples.

Figure S4. The O1s XPS spectra of aMnMOF and cMnMOF.

Figure S5. The XANES spectra of aMnMOF and the referenced compounds.

Figure S6. The phenol oxidase-like activity of cMnMOF in extended reaction system with high concentration and reaction time.

Figure S7. The phenol oxidase-like activity of different MnMOF.

Figure S8. Steady-state kinetic assays by using 2,4-DP as substrate. The Lineweaver–Burk plots of the double reciprocal of the Michaelis–Menten equation: (a) aMnMOF, (b) laccase.

Figure S9. The catalytic stability of aMnMOF under varied condition.

Figure S10. The normalized activity of aMnMOF and laccase along with storage time. Both of them were stored in a sealed bottle at room temperature.

Figure S11. The adsorption-desorption curves (a) and pore size distribution (b) of nanozymes prepared at atmospheric pressure.

Figure S12. The adsorption-desorption curves (a) and pore size distribution (b) of nanozymes prepared at high pressure.

Figure S13. The quenching experiments by using of β-carotene and semiquinone.

Fig S14. The GC-MS of intermediates generated during reaction.

Figure S15. The UV-vis curve of aMnMOF-TMB system, demonstrating its intrinsic oxidase-like activity.

Figure S16. The DFT model for aMnMOF (left) and cMnMOF (right). The Mn, C, and O atoms are represented by the purple, red, and gray color balls, respectively.

Figure S17. Optimized structures of hydroquinon adsorption on different MnMOFs, (a) aMnMOF, *E_ad_*=-0.48 eV, (b) cMnMOF, *E_ad_*=-0.71 eV. The Mn, C, and O atoms in MOF are represented by the purple, red, and gray color balls, respectively. The hydroquinon molecules are represented by green color.

Figure S18. DFT optimized structures of intermediates and transition states for the H abstraction from hydroquinon over aMnMOF (top) and cMnMOF (down). The Mn, C, and O atoms are represented by the purple, red, and gray color balls, respectively.

Figure S19. The EPR spectra of aMnMOF-dimeric compound system: (a) GGE, (b) PPE

Figure S20. The conversion rate of PPE and GGE by aMnMOF at 4 ^o^C and 37 ^o^C.

Figure S21. The UV-vis absorbance of dealkali lignin-aMnMOF system alongside reaction time.

Figure S22. The normalized efficiency of aMnMOF after different cycles.

Figure S23. The XRD pattern of aMnMOF before and after reaction.

Figure S24. The TEM images of aMnMOF after reaction.

Figure S25. The XPS pattern of Mn2p for aMnMOF before and after reaction.

Figure S26. The cell density of cyanobacteria cultured with: (a) different concentration of aMnMOF, (b) the supernatant of aMnMOF aqueous solution system after centrifugation.

Table S1. Catalytic performance comparison between aMnMOF and other laccase mimics.

| Nanozyme | Concentration (mg/mL) | *K_m_* (mM) | *V_max_* (mM s^-1^) | Ref |
| --- | --- | --- | --- | --- |
| This work | 0.08 | 0.33 | 0.708×10^−6^ | / |
| H–Mn–Cu NFs | 0.1 | 0.14±0.02 | 1.3±0.12×10^−6^ | 10 |
| Cu/H_3_-BTC MOF | 0.1 | 0.07 | 1.5×10^−6^ | 11 |
| CH–Cu | 0.1 | 0.58 | 0.045×10^−6^ | 12 |
| I-Cu | 0.15 | 0.172 | 0.410× 10^−6^ | 13 |
| Ru SA/GFs | 5 | 0.151 | 0.38 × 10^− 6^ | 14 |
| Ce-MOF-808 | 100 | 0.13 | 0.37×10^− 6^ | 15 |
| *β*-MnO_2_ | 0.2 | 0.99 | 1.14×10^− 6^ | 16 |

Table S2. Physic chemistry characteristic of different catalysts.

| Catalysts | Mn^2+^ % | Mn^3+^ % | Mn^4+^ % | Satellite peak % | AOS |
| --- | --- | --- | --- | --- | --- |
| cMnMOF | 51.93 | 27.62 | 9.18 | 11.28 | 2.33 |
| a-50(aMnMOF) | 35.06 | 36.39 | 22.45 | 6.09 | 3.45 |
| a-90 | 42.03 | 30.30 | 20.22 | 7.45 | 3.16 |
| a-120 | 48.34 | 28.72 | 15.65 | 7.30 | 2.69 |
| a-150 | 51.21 | 27.21 | 11.94 | 9.63 | 2.45 |
| h-90 | 43.19 | 29.70 | 19.78 | 7.34 | 2.81 |
| h-120 | 52.39 | 27.36 | 12.97 | 7.30 | 2.58 |
| h-150 | 49.05 | 26.55 | 12.68 | 11.82 | 2.42 |
| Note: AOS=8.956-1.126×*ΔE_Mn3s_.* *ΔE_Mn3s_* is the spacing between the Mn3s orbital partition fract. | | | | | |

Table S3. The comparison of performance between aMnMOF with nanocatalyst in PPE and GGE degradation.

| Catalyst | Type | Substrate | Reaction condition | Ref |
| --- | --- | --- | --- | --- |
| Ag_2_S@CdS | photo-catalysis | PPE | CH_3_CN, Ar (1 atm) and blue light | ^17^ |
| Pd/C | reductive depolymerization | GG-D and VG-D | H_2_, MeOH, 220 ^o^C | 18 |
| MoS_x_/Zn_3_In_2_S_6_ | photo-catalysis | PPE | Visible light, solvent  (CH_3_CN: H_2_O =1:1), vacuum K_2_S_2_O_8_ | 19 |
| Co@NCs | electro-catalysis | PPE | MeOH, 1 atm O_2_ and 110 ^o^C | 20 |
| Ru/C | reductive depolymerization | Multiple substrates | H_2_ (20 atm), MeOH and Cs_2_CO_3_ | 21 |
| Ni_3_S_2_ | pyrolysis | PPE | 260 ^o^C，2.0 MPa N_2_ and iPrOH | 22 |
| TiN | pyrolysis | PPE | 100 ^o^C, Ar (1 atm) and IPA | 23 |
| Pd-PdO/TiO_2_ | pyrolysis | PPE | 120 ^o^C, H_2_O, Ar | 24 |
| BetH_5_V_2_Mo_18_ | oxidation | PPE | 140 ^o^C, 0.8 Mpa O_2_ | 25 |
| Ni/Al_2_O_3_ | reductive depolymerization | PPE | Iso-propanol, 150 ^o^C | 26 |
| Pt_1_/N-CNTs | electro-catalysis | PPE | TBHP, nBu_4_NOH, MeCN and 20 mA | 27 |
| This work | catalytic oxidation | PPE and GGE | 4 ^o^C, atmosphere and neutral pH |  |

Table S4. The valance states of aMnMOF before and after reaction calculated from XPS data.

| aMnMOF | Mn^2+^ % | Mn^3+^ % | Mn^4+^ % | Satellite peak % |
| --- | --- | --- | --- | --- |
| Before reaction | 35.06 | 36.39 | 22.45 | 6.09 |
| After reaction | 34.41 | 37.22 | 22.37 | 6.00 |

Table S5. The FTIR features of dealkali lignin treated by aMnMOF

| Wavenumber (cm^-1^) | Characteristic peak spectrum |
| --- | --- |
| 2926 | C-H methyl |
| 1625 | conjugated carbonyl vibration with benzene ring |
| 1582 | C=C stretching of the aromatic ring |
| 1513 | C=C stretching of the aromatic ring |
| 1251 | C-O stretch (lignin) or C-O linkage in guaiacyl aromatic methoxyl groups |
| 1110 | C-O stretching vibration |
| 1090 | bending vibration in C-O and C-H |
| 870 | guaiacyl units |

Table S6. The parameters of raw kraft black liquor and after aMnMOF treatment.

| Parameters | Temperature  (^o^C) | Time (d) | | | | |
| --- | --- | --- | --- | --- | --- | --- |
|  |  | 0 | 5 | 10 | 15 | 20 |
| COD  (O_2_, mg/L) | 4 | 34675.2 | 20401.92 | 14192.64 | 10321.92 | 7741.44 |
|  | 20 |  | 22579.20 | 15644.16 | 9999.36 | 6693.12 |
|  | 37 |  | 17579.52 | 13547.52 | 7499.52 | 4273.92 |
| pH | 4 | 10.23 | 8.33 | 7.63 | 7.13 | 6.63 |
|  | 20 |  | 8.48 | 7.61 | 6.98 | 6.43 |
|  | 37 |  | 8.26 | 7.21 | 6.39 | 6.15 |
| Chroma (CU) | 4 | 17708.33 | 16562.50 | 13583.33 | 11062.50 | 9020.83 |
|  | 20 |  | 16041.67 | 12708.33 | 10895.83 | 8375.00 |
|  | 37 |  | 14729.17 | 9833.33 | 8312.50 | 5354.17 |
| Viscosity  (mPa·s) | 25 | 25 | 14.7 | 13.23 | 9.849 | 7.056 |

Table S7. The component of treated black liquor obtained from GC-MS measurement.

| Retention Time | Compound | 4 ℃ | 20 ℃ | 37 ℃ |
| --- | --- | --- | --- | --- |
| 7.007 | Heptanoic acid, 7,7-dimethoxy |  |  |  |
| 7.079 | 2-Ethoxyethanol | + | + | + |
| 7.14 | Octanal, pentadecafluoro |  |  |  |
| 7.265 | Trimethyl-(2-phenoxymethoxyethyl) | + | + | + |
| 7.4 | Propanedioic acid | + | + | + |
| 7.563 | 2-Phenoxy-1-propanol |  |  |  |
| 8.056 | 3-Methyl-2-ketobutyric acid eo-tms | + | + | + |
| 8.178 | Crotonic acid | + | + | + |
| 8.216 | Oxalic acid | + | + | + |
| 8.519 | Ethanamine | + | + | + |
| 8.664 | 2-Methylcyclohexylamine | + | + | + |
| 8.739 | Methyl aminoacetate | + | + | + |
| 9.137 | N-Methyl-N-(2-hydroxyethyl)carbamic acid, methyl ester | + | + | + |
| 9.287 | Citrulline | + | + | + |
| 9.425 | Ethylene glycol | + | + | + |
| 10.159 | 1,4-Dioxane-2,3-diol | + | + | + |
| 10.213 | Ethanol, 2-(2-ethoxyethoxy)-, acetate | + | + | + |
| 10.347 | Formic acid, 2-ethylbutyl ester | + | + | + |
| 10.522 | Halostachine | + | + | + |
| 10.573 | Thymol | + | + | + |
| 11.038 | Lactic Acid | + | + | + |
| 11.174 | Methylmalonic acid | + | + | + |
| 11.599 | 4-tert-Butoxybutan-1-ol | + | + | + |
| 11.976 | 3-Ethoxypropanol | + | + | + |
| 12.078 | 2-Ethoxyethanol | + | + | + |
| 12.322 | 2-Hydroxybutyric acid | + | + | + |
| 13.301 | 3-Methylphenylacetic acid trimethylsilyl ester | + | + | + |
| 13.389 | 2-(2-Methoxyethoxy)acetic acid | + | + | + |
| 14.812 | Glycerol | + | + | + |
| 15.601 | 2-Methyl-6-tert-butylphenol | + | + | + |
| 15.828 | Diethylene glycol | + | + | + |
| 18.657 | Eicosane | + | + | + |
| 19.839 | 4-Hydroxybenzoic acid | + | + | + |
| 24.504 | Palmitic Acid | + | + | + |
| 29.53 | 1-Monopalmitin | + | + | + |

Table S8. A comparison between aMnMOF and other system in black liquor treatment.

| Microbes | Reaction temperature (^o^C) | Reaction time (day) | Removal efficiency (%) | | | | Ref |
| --- | --- | --- | --- | --- | --- | --- | --- |
|  |  |  | Lignin | pH | COD | Chroma |  |
| aMnMOF | 4 | 10 | 83.63 | 10.23 to 6.63 | 77.67 | 49.42 | This work |
|  | 37 | 10 | 87.07 | 10.23 to 6.15 | 87.67 | 69.98 |  |
| S. marcescens, Klebsiella pneumoniae, Citrobacter sp. | 40 | 8 | / | / | 83 | 85 | 28 |
| Halomonas sp. | 37 | 5 | / | 11 to 7.8 | / | 51 | 29 |
| Arthrobacter sp. C2 | 15 | 15 | 65.5 |  | 64.2 | 96.4 | 30 |
| P. chrysosporium | 25 | 10 | / | / | 65 | 37 | 31 |
| A. uvarum | 25 | 10 | / | / | 61 | 81 | 32 |
| Dipodasceus australiansis M-2. | 30 | 8 | 89 | / | / | 41 | 33 |
| Serratia sp. AXJ-M | 50 | 7 | 60 | / | 80 | 80 | 34 |
| PAC+Fenton-like method (FeCl_3_.6H_2_O and H_2_O_2_) | 50 | 1 | 94-98 | / | / | 70 | 35 |
| Fe_3_O_4_+ *Pseudomonas otitidis* | / | / | 70 | / | 52 | 64 | 36 |
| MnO_2_+H_2_O_2_+light | / | / | / | 11 to 9.8 | 75 | / | 37 |

**Reference**

[1]	J. VandeVondele, M. Krack, F. Mohamed, et al., Quickstep: Fast and accurate density functional calculations using a mixed Gaussian and plane waves approach. [*J*]. Comput. Phys. Commun. **2005,** 167 (2), 103-128.

[2]	S. Goedecker, M. Teter.J. Hutter, Separable dual-space gaussian pseudopotentials. [*J*]. Phys. Rev. B **1996,** 54 (3), 1703-1710.

[3]	C. Hartwigsen, S. Goedecker.J. Hutter, Relativistic separable dual-space gaussian pseudopotentials from H to Rn. [*J*]. Phys. Rev. B **1998,** 58 (7), 3641-3662.

[4]	M. Krack.M. Parrinello, All-electron ab-initio molecular dynamics. [*J*]. Phys. Chem. Chem. Phys. **2000,** 2 (10), 2105-2112.

[5]	J. VandeVondele.J. Hutter, Gaussian basis sets for accurate calculations on molecular systems in gas and condensed phases. [*J*]. J. Chem. Phys. **2007,** 127 (11), 114105.

[6]	J.P. Perdew, K. Burke.M. Ernzerhof, Generalized gradient approximation made simple. [*J*]. Phys. Rev. Lett. **1996,** 77, 3865.

[7]	S. Grimme, J. Antony, S. Ehrlich, et al., A consistent and accurate ab initio parametrization of density functional dispersion correction (DFT-D) for the 94 elements H-Pu. [*J*]. J. Chem. Phys. **2010,** 132 (15), 154104.

[8]	G. Henkelman, B.P. Uberuaga.H. Jonsson, A climbing image nudged elastic band method for finding saddle points and minimum energy paths. [*J*]. J. Chem. Phys. **2000,** 113 (22), 9901-9904.

[9]	G. Mills, H. Jonsson.G.K. Schenter, Reversible work transition-state theory - application to dissociative adsorption of hydrogen. [*J*]. Surf. Sci. **1995,** 324 (2-3), 305-337.

[10]	X. Wu, S. Xie, C. Liu, et al., Ligand-controlled photocatalysis of CdS quantum dots for lignin valorization under visible light. [*J*]. ACS Catal. **2019,** 9 (9), 8443-8451.

[11]	H. Li.G. Song, Paving the way for the lignin hydrogenolysis mechanism by deuterium-incorporated *β*-O-4 mimics. [*J*]. ACS Catal. **2020,** 10 (20), 12229-12238.

[12]	L. Jing, M. Xie, Y. Xu, et al., Multifunctional 3D MoSx/Zn3In2S6 nanoflower for selective photothermal-catalytic biomass oxidative and non-selective organic pollutants degradation. [*J*]. Appl. Catal. B-Environ. **2022,** 318, 121814.

[13]	K. Sun, S. Chen, J. Zhang, et al., Cobalt nanoparticles embedded in N-doped porous carbon derived from bimetallic zeolitic imidazolate frameworks for one-pot selective oxidative depolymerization of lignin. [*J*]. ChemcatChem **2019,** 11 (4), 1264-1271.

[14]	H. Li.G. Song, Ru-catalyzed hydrogenolysis of lignin: base-dependent tunability of monomeric phenols and mechanistic study. [*J*]. ACS Catal. **2019,** 9 (5), 4054-4064.

[15]	C. Chen, D. Wu, P. Liu, et al., Eco-friendly preparation of ultrathin biomass-derived Ni3S2-doped carbon nanosheets for selective hydrogenolysis of lignin model compounds in the absence of hydrogen. [*J*]. Green Chem. **2021,** 23 (8), 3090.

[16]	Z. Liu, Y. Huang, G. Xiao, et al., Visible-light-driven efficient cleavage of *β*-O-4 linkage in a lignin model compound: phenethyl phenyl ether photocatalyzed by titanium nitride nanoparticles. [*J*]. Energy Fuel. **2021,** 35 (16), 13315-13324.

[17]	Z. Dou, Z. Zhang.M. Wang, Self-hydrogen transfer hydrogenolysis of native lignin over Pd-PdO/TiO_2_. [*J*]. Appl. Catal. B-Environ. **2022,** 301, 120767.

[18]	Z. Li, Y. Li, Y. Chen, et al., Developing dawson-type polyoxometalates used as highly efficient catalysts for lignocellulose transformation. [*J*]. ACS Catal. **2022,** 12 (15), 9213-9225.

[19]	L. Jiang, H. Guo, C. Li, et al., Selective cleavage of lignin and lignin model compounds without external hydrogen, catalyzed by heterogeneous nickel catalysts. [*J*]. Chem. Sci. **2019,** 10 (16), 4458-4468.

[20]	L. Ma, H. Zhou, X. Kong, et al., An electrocatalytic wtrategy for C-C bond cleavage in lignin model compounds and lignin under ambient cConditions. [*J*]. Acs Sustain. Chem Engin. **2021,** 9 (4), 1932-1940.

[21] H. Li, G. Song, Ru-catalyzed hydrogenolysis of lignin: base-dependent tunability of monomeric phenols and mechanistic study. ACS Catal., 2019, 9, 4054.

[22] C. Chen, D. Wu, P. Liu, J. Li, J. Jiang, Eco-friendly preparation of ultrathin biomass-derived Ni3S2-doped carbon nanosheets for selective hydrogenolysis of lignin model compounds in the absence of hydrogen. Green Chem. 2021, 23, 3090.

[23] Z. Liu, Y. Huang, G. Xiao, P. Li, H. Su, S. Sarina, H. Zhu, Visible-light-driven efficient cleavage of *β*-O-4 linkage in a lignin model compound: phenethyl phenyl ether photocatalyzed by titanium nitride Nanoparticles. Energ. Fuel. 2021, 35, 13315.

[24] Z. Dou, Z. Zhang, M. Wang, Self-hydrogen transfer hydrogenolysis of native lignin over Pd-PdO/TiO_2_ [J]. Appl. Catal. B-Enviro. 2022, 301, 120767.

[25] Z. Li, Y. Li, Y. Chen, Q. Wang, M. Jadoon, X. Yi, X. Duan, X. Wang, Developing dawson-type polyoxometalates used as highly efficient catalysts for lignocellulose transformation. ACS Catal, 2022, 12, 9213.

[26] L. Jiang, H. Guo, C. Li, P. Zhou, Z. Zhang, Selective cleavage of lignin and lignin model compounds without external hydrogen, catalyzed by heterogeneous Nickel Catalysts. Chem. Sci, 2019, 10, 4458.

[27] L. Ma, H. Zhou, X. Kong, Z. Li, H. Duan, An electrocatalytic strategy for C-C bond cleavage in lignin model compounds and lignin under ambient conditions. Acs Sustain. Chem. Eng. 2021, 9, 1932.

[28] Chandra, R.; Abhishek, A.; Sankhwar, M., Bacterial decolorization and detoxification of black liquor from rayon grade pulp manufacturing paper industry and detection of their metabolic products. Biores. Technol. 2011, 102 (11), 6429-6436.

[29] Yang, C.; Niu, Y.; Su, H., et al., A novel microbial habitat of alkaline black liquor with very high pollution load: Microbial diversity and the key members in application potentials. Biores. Technol. 2010, 101 (6), 1737-1744.

[30]	Chandra, R.; Abhishek, A., Bacterial decolorization of black liquor in axenic and mixed condition and characterization of metabolites. Biodegradation 2011, 22, 603-611.

[31]	Wang, Y.; Chen, X.; Wu, B., et al., Potential and mechanism for bioremediation of papermaking black liquor by a psychrotrophic lignin-degrading bacterium, Arthrobacter sp. C2. J. Hazard. Mater. 2022, 439, 129534.

[32] Díaz, A.I.; Laca, A.; Lima, N., et al., Treatment of kraft black liquor using basidiomycete and ascomycete fungi. Process. Saf. Environ. 2022, 168, 67-76.

[33] Parveen, S.; Ali, M.I.; Aslam, M., et al., Optimizing biocatalytic potential of Dipodascus australiensis M-2 for degrading lignin under laboratory conditions. Microbio. Res. 2022, 265, 127179.

[34]	An, X.; Zhong, B.; Chen, G., et al., Evaluation of bioremediation and detoxification potentiality for papermaking black liquor by a new isolated thermophilic and alkali-tolerant Serratia sp. AXJ-M. J. Hazard. Mater. 2021, 406, 124285.

[35]	M. Muryanto, A.A. Sari.H. Abimanyu, Degradation of black liquor from bioethanol process using coagulation and Fenton-like methods. AIP Conf. Proc. 2017, 1803, 020007.

[36]	E. El-Bestawy, S.W.M. Hassan.A.A. Mohamed, Enhanced biodegradation of lignin and lignocellulose constituents in the pulp and paper industry black liquor using integrated magnetite nanoparticles/bacterial assemblage. Applied Water Science 2024, 14 (10), 211.

[37]	A.G.B. Dileepan.S. Jeyaram, Sustainable industrial wastewater via photocatalysis using green-synthesised MnO_2_ nanoparticles. Mater. Res. Innova. 2025, 1(26), 2534542.
